# Successful observation of intraductal papillary mucinous neoplasm in the accessory pancreatic duct using a novel peroral pancreatoscopy

**DOI:** 10.1055/a-2371-1009

**Published:** 2024-08-07

**Authors:** Jun Noda, Yuichi Takano, Masataka Yamawaki, Tetsushi Azami, Fumitaka Niiya, Fumiya Nishimoto, Masatsugu Nagahama

**Affiliations:** 1 26858 Division of Gastroenterology, Department of Internal Medicine, Showa University Fujigaoka Hospital, Yokohama, Japan

The utility of a peroral pancreatoscopy (POPS) system for intraductal papillary mucinous neoplasm (IPMN) has been widely reported 1 . However, accessing the accessory pancreatic duct using POPS from the major papilla is considered challenging due to the steep angle. We successfully employed a novel POPS system (eyeMax; Microtech Endoscopy, Nanjing, China) ( Fig. 1 ) for the observation and biopsy of an IPMN in the accessory pancreatic duct from the major papilla.

**Fig. 1:**
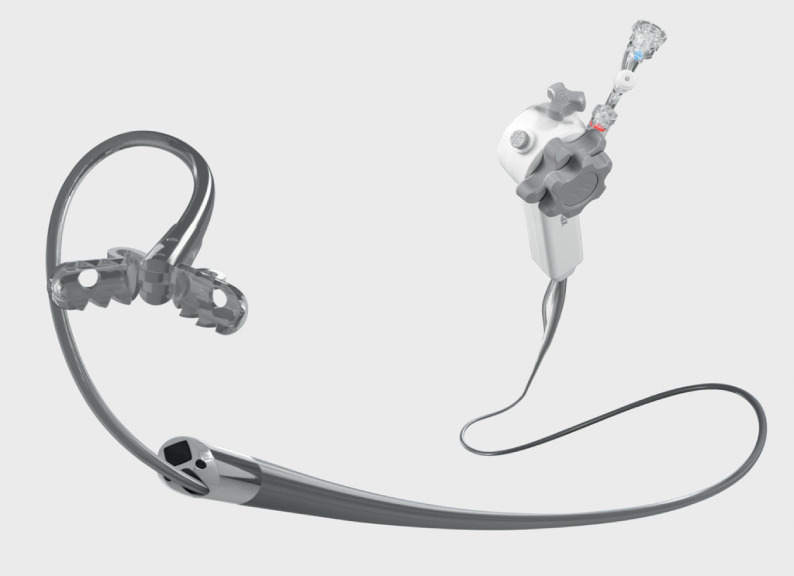
The novel peroral pancreatoscopy system with a diameter of 9 French (Fr). A biopsy forceps with an outer diameter of 1.0 mm. Source: MC Medical.

A 70-year-old woman had a main pancreatic duct dilatation detected on abdominal ultrasound during a health check. Contrast-enhanced computed tomography revealed a 10-mm tumor in the accessory pancreatic duct ( Fig. 2 ). Endoscopic ultrasound (EUS) revealed a papillary tumor with contrast enhancement using Sonazoid within the accessory pancreatic duct. Endoscopic retrograde cholangiopancreatography (ERCP) was performed. After pancreatic duct cannulation, endoscopic pancreatic sphincterotomy was performed, and a guidewire (VisiGlide2; Olympus, Tokyo, Japan) was carefully manipulated into the accessory pancreatic duct from the main papilla. A 9-Fr pancreatic pancreatoscope was inserted through the major papilla. The pancreatoscope was tilted steeply upward and inserted into the accessory pancreatic duct in an inverted position. We successfully observed the reddish papillary tumor. We performed three biopsies of the tumor ( Video 1 ). Although no horizontal extension towards the tail was observed, a negative biopsy was performed at the confluence with the accessory pancreatic duct. The procedure was completed with the insertion of a 6-Fr α endoscopic nasopancreatic drainage tube. Mild hyperamylasemia was noted, but no major complications occurred. Pathological examination revealed a papillary tumor growth with atypical mucinous cells, which was diagnosed as intraductal papillary mucinous carcinoma (IPMC) ( Fig. 3 ); a pancreaticoduodenectomy was planned.

**Fig. 2:**
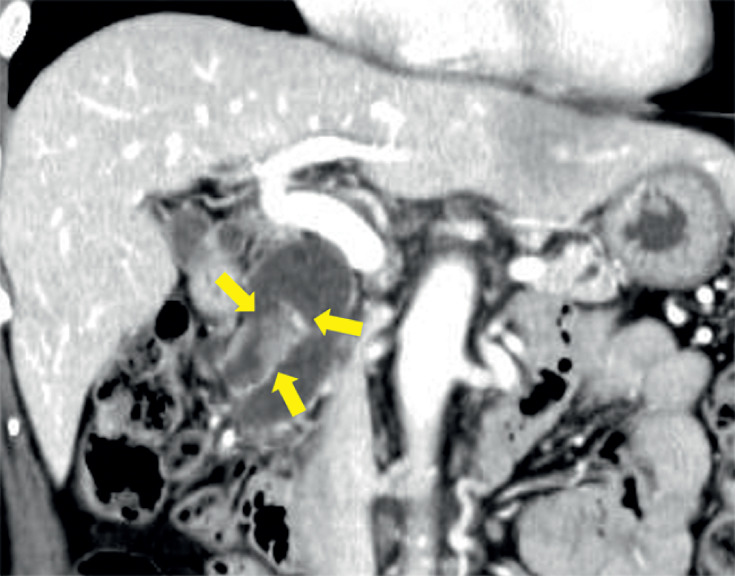
Contrast-enhanced computed tomography revealed a 10-mm tumor in the accessory pancreatic duct.

**Fig. 3:**
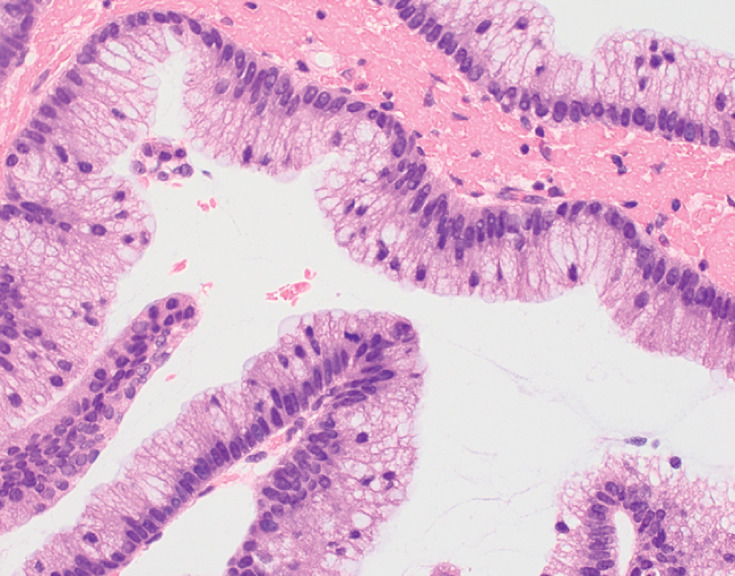
Pathological examination revealed a papillary tumor growth with atypical mucinous cells, which diagnosed as intraductal papillary mucinous carcinoma.

We successfully observed and biopsied an intraductal papillary mucinous neoplasm in the strongly curved sub-pancreatic duct area using a novel peroral pancreatoscopy system.Video 1

When an approach to a strongly curved pancreatic duct or the accessory pancreatic duct is necessary, this pancreatoscope allows smooth insertion due to its high flexibility and can provide high quality images.

Endoscopy_UCTN_Code_TTT_1AR_2AD

## Notes

Endoscopy E-Videos https://eref.thieme.de/e-videos
*E-Videos* is an open access online section of the journal *Endoscopy* , reporting on interesting cases and new techniques in gastroenterological endoscopy. All papers include a high-quality video and are published with a Creative Commons CC-BY license. Endoscopy E-Videos qualify for HINARI discounts and waivers and eligibility is automatically checked during the submission process. We grant 100% waivers to articles whose corresponding authors are based in Group A countries and 50% waivers to those who are based in Group B countries as classified by Research4Life (see: https://www.research4life.org/access/eligibility/ ). This section has its own submission website at https://mc.manuscriptcentral.com/e-videos .

## References

[1] Ohtsuka T Gotoh Y Nakamura M Role of SpyGlass-DStm in the preoperative assessment of pancreatic intraductal papillary mucinous neoplasm involving the main pancreatic duct Pancreatology 2018 18 566 571 29730245 10.1016/j.pan.2018.04.012

